# Impaired ATG16L-Dependent Autophagy Promotes Renal Interstitial Fibrosis in Chronic Renal Graft Dysfunction Through Inducing EndMT by NF-κB Signal Pathway

**DOI:** 10.3389/fimmu.2021.650424

**Published:** 2021-04-13

**Authors:** Zeping Gui, Chuanjian Suo, Zijie Wang, Ming Zheng, Shuang Fei, Hao Chen, Li Sun, Zhijian Han, Jun Tao, Xiaobin Ju, Haiwei Yang, Min Gu, Ruoyun Tan

**Affiliations:** ^1^ Department of Urology, The First Affiliated Hospital of Nanjing Medical University, Nanjing, China; ^2^ Department of Urology, The Second Affiliated Hospital of Nanjing Medical University, Nanjing, China

**Keywords:** chronic renal graft dysfunction, renal interstitial fibrosis, ATG16L, autophagy, EndMT, inflammatory cytokines

## Abstract

Chronic renal graft dysfunction (CAD) is caused by multiple factors, including glomerular sclerosis, inflammation, interstitial fibrosis and tubular atrophy (IF/TA). However, the most prominent elements of CAD are IF/TA. Our studies have confirmed that endothelial-mesenchymal transition (EndMT) is an important source to allograft IF/TA. The characteristic of EndMT is the loss of endothelial marker and the acquisition of mesenchymal or fibroblastic phenotypes. Autophagy is an intracellular degradation pathway that is regulated by autophagy-related proteins and plays a vital role in many fibrotic conditions. However, whether or not autophagy contributes to fibrosis of renal allograft and how such mechanism occurs still remains unclear. Autophagy related 16 like gene (ATG16L) is a critical autophagy-related gene (ARG) necessary for autophagosome formation. Here, we first analyzed kidney transplant patient tissues from Gene Expression Omnibus (GEO) datasets and 60 transplant patients from our center. Recipients with stable kidney function were defined as non-CAD group and all patients in CAD group were histopathologically diagnosed with CAD. Results showed that ATG16L, as one significant differential ARG, was less expressed in CAD group compared to the non-CAD group. Furthermore, we found there were less autophagosomes and autolysosomes in transplanted kidneys of CAD patients, and downregulation of autophagy is a poor prognostic factor. In vitro, we found out that the knockdown of ATG16L enhanced the process of EndMT in human renal glomerular endothelial cells (HRGECs). *In vivo*, the changes of EndMT and autophagic flux were then detected in rat renal transplant models of CAD. We demonstrated the occurrence of EndMT, and indicated that abundance of ATG16L was accompanied by the dynamic autophagic flux change along different stages of kidney transplantation. Mechanistically, knockdown of ATG16L, specifically in endothelial cells, reduced of NF-κB degradation and excreted inflammatory cytokines (IL-1β, IL-6 and TNF-α), which could facilitate EndMT. In conclusion, ATG16L-dependent autophagic flux causing by transplant showed progressive loss increase over time. Inflammatory cytokines from this process promoted EndMT, thereby leading to progression of CAD. ATG16L served as a negative regulator of EndMT and development of renal graft fibrosis, and autophagy can be explored as a potential therapeutic target for chronic renal graft dysfunction.

## Introduction

Kidney transplantation is one of the optimal treatment for patients with uremia because it significantly improves their quality of life (1). However, there is a relatively high number of renal allograft dysfunction after transplantation due to the chronic progressive deterioration of renal function, which is a key factor affecting the long-term survival of transplanted kidney (2). Chronic renal graft dysfunction (CAD), formerly known as chronic allograft nephropathy, is a multifactorial condition associated with progressive renal interstitial fibrosis. Various factors are known to contribute to the loss of renal allograft function, including, but not limited to, acute and chronic rejection, ischemia and reperfusion inflammatory and tissue rebuilding process, and drugs-related nephrotoxicity (3).

CAD is morphologically characterized by inflammation, progressive interstitial fibrosis and tubular atrophy(IF/TA), and glomerular sclerosis (4). Among them, IF/TA is key factor determining renal allograft function, but the mechanisms by which it occurs are still unknown. Several factors have been identified to contribute to the high proportion of renal allograft loss. In our previous study, we also confirmed that the main pathological process of CAD was renal allograft IF/TA, which is characterized by excessive deposition of extracellular matrix in transplanted renal tubular and interstitial tissue (5). Studies have verified that collagens are mainly secreted by myofibroblasts, and collagens secretion lead to extracellular matrix sedimentation and subsequently transplant kidney IF/TA. There are four principal cells involved in the formation of myofibroblasts: epithelial cells, endothelial cells, bone marrow-derived fibroblasts and microvascular pericytes (6, 7). They play a crucial role in repairing and protecting integrity of kidney tissue. Among them, external stimuli enable differentiation of intrinsic kidney cells such as epithelial cells to myofibroblasts, which can produce extracellular matrix. This process is called epithelial-to-mesenchymal transition (EMT). EMT usually involved three types: (I) embryogenesis, (II) tissue repair and fibrosis, (III) metastasis (8). Several studies have been done to elucidate the molecular and cellular mechanisms of type II EMT in organ fibrosis (9, 10), however, roles of EMT in different primary fibroblast-generating process during the CAD progression still remain inconclusive.

With kidney transplantation, there is always an increased likelihood of damage to the allograft macro- and microvasculature, due to the physical location of the allograft endothelium that makes it an initial target of choice for allograft injury (11). Allografts act as potential targets for immune response mediated by quite a number of serum inflammatory cytokines. Studies indicated that many inflammatory cytokines involved in CAD process (12) and endothelial cells at sites of renal allografts were not only participants in inflammation but also regulators of inflammation (11). Our previous research revealed that TNF‐α more highly expressed in CAD group than non-CAD group. TNF‐α also facilitated the EMT process in human proximal tubular cells (HK_2_) (13). Given the intimate contact between endothelial cells and blood, circulating inflammatory cells and cytokines will first attack the allograft endothelium. Chronic stimulation from inflammatory cytokines leads to endothelial cells undergoing disorders of cell structure and internal environment, which results in endothelial injury. Emerging studies suggest that endothelial injury contributes to extracellular matrix deposition and plays a key role in the organ fibrosis diseases. Recently, Endothelial-to-mesenchymal transition (EndMT) is considered as the principal cause of the endothelial injury and promotes the progress of IF/TA during renal fibrosis disease (5, 9). EndMT is a distinctive type of EMT. It is characterized by cells that gradually lose endothelial markers, such as CD31 and CD34, and gain mesenchymal or myofibroblastic phenotype, such as a-smooth muscular actin (α-SMA), collagen I, and fibronectin (FN). The regulatory mechanism for EndMT remains a complex issue. The occurrence of EndMT could be affected by many factors such as oxidative stress, hypoxic and various injuries (14, 15). But almost all of these regulatory factors eventually come together in one direct action, which is inflammatory cytokines. In our previous studies, we showed that the EndMT was an important factor in the pathogenesis of IF/TA and CAD through the TGF-β/Smad signaling pathways (5). In addition, still other scholars suggested that TGF-β expression was up-regulated by TNF‐α (16). So, inflammatory cytokines such as TNF‐α may also be an important pathogenic factor in vascular endothelial injury that characterizes CAD progression. Although the initial factors of EndMT were different in various microenvironments, the final outcome was the same. Hence, it sufficed to hypothesize that there must be a central regulatory mechanism in the production of inflammatory cytokines and progression of EndMT.

Autophagy is a cellular pathway responsible for protein and organelle degradation (17). The process of autophagy is also known as autophagic flux, and the strength of this process often represents the degree of activation of autophagy. Autophagy is involved in different renal pathophysiological processes, including glomerulosclerosis, diabetic nephropathy and cystic kidney disease (18). Some studies reported that autophagy was a cytoprotective process. A classic defense for this presupposition is as seen in mice with proximal tubule ATG5 deletion where the deletion promoted more severe renal fibrosis due to impaired autophagy (19). Other studies have also found that proximal tubule epithelial cells conditional knockdown ATG5 aggravated acute kidney injury in the early stage, but decelerated the progression of kidney fibrosis in the recovery or repair process (20). However, the specific interaction between autophagy and EMT or EndMT on fibrosis disease still remains controversial. Several studies suggested that autophagy inhibition could induce EMT and fibrosis by affecting the aberrant epithelial–fibroblast crosstalk in idiopathic pulmonary fibrosis (21). Others showed that Rapamycin promotes EndMT through the activation of autophagy during premature senescence (22). ATG16L gene mainly includes ATG16L1 and ATG16L2. ATG16L2 homo- and hetero-oligomerizes with ATG16L1. But ATG16L2 has less associated with autophagy (23). ATG16L1 has been identified as an important autophagy-related gene, promotes autophagosome formation at the plasma membrane and plays a critical role in the lipidated form of LC3 (23). Here, ATG16L1 is collectively referred to as ATG16L. As a key factor in autophagy, ATG16L was once reported that was related to the pathogenesis of several inflammatory diseases. For instance, the mutation of ATG16L conferred a strong predisposition to Crohn’s disease development (24). However, the mechanism of ATG16L action and autophagy has not yet been well-studied in a targeted approach such as in the management and prognostication of kidney transplantation. So the dynamic role of autophagic flux and ATG16L-dependent autophagy must be the focus of research for potential therapeutics in CAD progression.

In this study, we explored the potential role of ATG16L-dependent autophagy in CAD progression. We revealed that the loss of ATG16L and autophagy were associated with occurrence of EndMT in clinical CAD patients and kidney transplanted rats. We then found that the levels of EndMT and renal allograft interstitial fibrosis were enhanced after altering autophagy activity by knocking down ATG16L gene. In addition, potential underlying mechanisms were also explored by RNA sequencing, we found that impairment of ATG16L contributed to the nuclear factor-κB (NF-κB) pathway activation and the inflammatory cytokines secretion, then promoted EndMT progression.

## Materials and Methods

### Ethics Statement

The study’s protocols complied with the Declaration of Helsinki and Istanbul. Human studies were examined and authorized by the local ethics committee of the First Affiliated Hospital of Nanjing Medical University (ID: IACUC-2010020). Informed consent was acquired from all transplant recipients as well as nephrectomy patients who were included in this research.

The studies involving rats were examined and authorized by the local ethics committee of the First Affiliated Hospital of Nanjing Medical University (ID: IACUC-2010020). Written informed consent is given by the owners for the participation of their animals in this research.

### Sample Collection

Sixty adults who had received living or deceased donor kidney transplants started to be followed up from January 2010 to December 2017 at First Affiliated Hospital of Nanjing Medical University. Patients with serum creatinine level consistently < 141.46 μmol/L (1.6 mg/dl) for at least 12 months after kidney transplantation and no other complications such as episodes of significant rejection, drug toxicity injury, and infection were assigned to the non-CAD group. Patients in CAD group were defined as elevated serum creatinine greater than or equal to 141.46 μmol/L (1.6 mg/dl) for at least three months, and they were diagnosed by two independent pathology experts combined with biopsy results, laboratory indexes and imaging features. Adjacent normal kidney tissues were obtained from regions outside the tumor margin (>5cm) in patients with radical nephrectomy operations. Blood samples were obtained after patients had given informed consent. The specific step can be referred to our previous studies (5). The baseline characteristics of patients in the CAD group and non-CAD group were as shown in **Table 1**.

**Table 1:** Baseline characteristics of the CAD and non-CAD groups.

Clinical variables	CAD group	non-CAD group	P Value
**Case number (n)**	30	30	NS
**Age (years, mean ± SD)**	35.59 ± 3.09	38.61 ± 2.41	NS
**Gender (Male/Female)**	18/12	20/10	NS
**BMI (kg/m2, mean ± SD)**	22.64 ± 4.71	23.21 ± 4.2	NS
**Transplant duration (years, range)**	9.1 (6.5-13)	3.4 (2.3-4.8)	<0.001
**Primary/secondary transplant**	30/0	30/0	NS
**PRA before renal transplant (%)**	0	0	NS
**Donor source**			NS
**Living-related**	25	21	
**Cadaveric**	5	9	
**Immunosuppressive regimen**			NS
**Prednisone + MMF + Tac**	20	22	
**Prednisone + MMF + CsA**	10	8	
**Biochemical parameters**			
**Serum creatinine** **(μmol/L, mean ± SD)**	418.1 ± 20.8	93.37 ± 10.68	<0.001
eGFR* (min/1.73 m^2^, mean ± SD)	24.71± 2.88	76.79 ± 6.23	<0.001

SD, standard deviations; NS, no significance; CAD, Chronic allograft dysfunction; BMI, body mass index; PRA, panel reaction antibody; MMF, mycophenolate mofetil; CsA, cyclosporine A; Tac, tacrolimus; eGFR, estimated glomerular filtration rate; SD, standard deviation. *eGFR was estimated by the Cockcroft-Gault formula: eGFR = (140-age) x weight/72 x serum creatinine x (0.85 if female).

### Rats and Animals Models

Adult male F344 and Lewis rats (Weight 250 ± 10.3 g) were obtained from Charles River Laboratories (Beijing, China) and abided by the guidelines of the Institutional Animal Care and Use Committee at Nanjing Medical University. The animals were handled in accordance with the norms of Nanjing Medical University and the guidelines published by the US National Institutes of Health.

All these animals experienced orthotopic left kidney transplantation. Lewis rats were used as recipients and syngeneic donors (Syn group), F344 rats as allogeneic donors (Allo group). The right kidney was excised simultaneously. The average time of cold ischemia was less than 20 minutes and warm ischemia was less than 35 minutes. Cyclosporine A (5 mg/kg, qd, ip; Neoral, Novartis, Switzerland) was used for 14 days to avoid the acute rejection,

### Pharmaceutical Treatment and Tissue Harvest

The transplant kidneys from rats were harvested at weeks 4, 8, 12 and 16 after surgery. Paraffin-embedded formalin-fixed (10% neutral formalin) renal allograft tissue specimens were obtained for histological and IHC/IF staining. Remaining kidney stored at − 80°C refrigerator for detection of RNA and protein.

### HE and Masson Trichrome Staining Assay

Protocals of HE and Masson trichrome staining assay can be referred to our previous studies (5). To identify the severity of CAD and the extent of fibrotic area, the renal allograft fibrosis area was quantified with Masson trichrome staining. For each slice, five random visual fields under × 400 microscope were selected. Area positive for Masson trichrome was measured by two pathologists blinded to the experimental design using the Image‐Pro Plus (Media Cybernetics, Rockville, MD).

### Immunohistochemistry Staining Assay

Kidney tissues fixed with formalin were cut into 3 μm thick paraffin sections. The process of deparaffinized, hydrated, and antigen-retrieved were consistent with our previous studies (5). The antibodies [anti‐ATG16L (1:100; Abcam, USA), anti‐α‐SMA (1:200; Abcam, USA), anti‐Fibronectin (1:100; Abcam, USA) and anti‐CD31 (1:100; CST, USA)] were incubated overnight at 4°C after sections blocked with 10% normal donkey serum. Next steps were also performed as described earlier with biotinylated goat anti‐mouse/rabbit IgG (0.5 μg/mL; Abcam) and substrate 3-amino-9-ethylcarbazole or 3,3′diaminobenzidine (Vector Laboratories, Burlingame, CA). The stained slides were photographed using a Nikon Eclipse 80i microscope equipped with a digital camera (DS-Ri1, Nikon, Shanghai, China).

### Indirect Immunofluorescence Staining Assay

After fixing with 4% formaldehyde solution, renal allograft sections and cell climbing slices were penetrated with 0.1% Triton X-100 for 1 h, then blocked with 5% goat serum for 1 h. Afterward, sections and cell climbing slices were incubated with the anti‐ NF-κB p65 (1:200; Abcam, USA) at 4°C overnight. Incubation conditions of secondary antibody Dapi can refer to our previous studies (5). Slides were viewed with a Nikon Eclipse 80i fluorescence microscope (DS-Ri1, Nikon, Shanghai, China).

### Autophagic Flux Detection

AAV-mRFP-GFP-LC3 (Hanbio, Shanghai, China) was stereotactically injected into left kidney of Lewis rats (3 μL) before 14 days before transplantation. The kidney was removed at 4, 8, 12, 16 weeks after transplantation and fixed with 4% paraformaldehyde for 24 h. Slides (30 μm) were viewed with a Nikon Eclipse 80i fluorescence microscope (DS-Ri1, Nikon, Shanghai, China). Yellow spots indicate autophagosomes and red spots indicate autolysosomes. When red signal stronger than the yellow signal represent autophagic flux is activated. When more yellow signal than red signal represent autophagic flux is impaired.

### Electron Microscopy

The samples were fixed with ice-cold glutaraldehyde (3% in 0.1 M cacodylate buffer, pH 7.4) and further processed by the Core Facility (Servicebio, Wuhan, China). Observation was performed on a JEOL JEM-2100 transmission electron microscope.

### Renal Function Detection

We used a rat QuantiChrom Creatinine Assay Kit and QuantiChrom Urea Assay Kit (Jiancheng, Beijing, China) to detected the concentrations of rat blood creatinine and urea nitrogen according to the manufacturer’s instructions.

### Uria Protein Detection

The 24 h urine samples were collected using metabolic cages, and urinary protein excretion was tested with the commercial kit (Mlbio, Shanghai, China) according to the instructions of the manufacturer.

### Real-Time PCR Assessment

Briefly, total RNA was purified from HRGECs using the RNA extraction kits (TIANGEN, Beijing, China). cDNA (cDNA) was synthesized as described previously (5). Gene expression was measured by real-time PCR assay (Vazyme) and a DNA Engine Opticon 2 System (BioRad laboratories, Hercules, CA). The primer sequences were described as follows:

IL-1 β: 5′‐ TTCCTGTTGTCTACACCAATGC‐3′ (F)
5′‐CGGGCTTTAAGTGAGTAGGAGA‐3′ (R);
IL-6: 5′‐TCTCTCCGCAAGAGACTTCCA‐3′ (F)
5′‐ ATACTGGTCTGTTGTGGGTGG‐3′ (R);
TNF-α: 5′‐ CCTCTCTCTAATCAGCCCTCTG‐3′ (F)
5′‐ GAGGACCTGGGAGTAGATGAG ‐3′ (R);
Actin: 5′‐ TGACGTGGACATCCGCAAAG‐3′ (F)
5′‐ CTGGAAGGTGGACAGCGAGG‐3′ (R);

### Western Blot and Elisa Assay

Briefly, proteins from cells and tissues were extracted in RIPA buffer (Thermo ScientificTM, Chelmsford, MA, USA) containing phosphatases and proteases inhibitor cocktails (Sigma, St Louis, MO, USA). Proteins (20 μg) were transferred to a PVDF membrane (Millipore, IPVH00010, Massachusetts, USA) following SDS-PAGE. The slides were incubated in blocking solution (5% non-fat milk) for 60 min, and then PVDF membrane was incubated with primary antibody without washing in cold room overnight. After that, the process of PVDF membrane wash by tris buffered saline-tween (TBST) buffer, incubated by secondary antibody, ECL tableting and exposed were consistent with our previous studies (5). The primary antibodies were listed as follows: anti‐GAPDH (1:1000; CST, USA), anti‐CD31 (1:1000; CST, USA), anti‐α‐SMA (1:1000; Abcam, USA), anti‐fibronectin (1:1000; BD Biosciences, USA), anti‐LC3 (1:1000; CST, USA), anti‐ATG16L (1:1000; CST, USA), anti‐NF-κB p65 (1:1000; CST, USA), anti‐ Phospho-NF-κB p65 (1:1000; CST, USA). Quantification was performed by measuring the intensity of the signals with the aid of NIH image analysis software. MAP1LC3 was detected using an ELISA kit (ml000829, Mlbio, China) according to the manufacturer’s instruction.

### Cell Culture, Treatment, and shRNA Transfection

Human renal glomerular endothelial cells (HRGECs) were cultured in Endothelial cell medium (ECM, ScienCell Research Laboratories Carlsbad, CA, USA) containing 5% fetal bovine serum. Equipment Setup CO2 Incubator Culture HRGECs in a humidified atmosphere containing 5% CO2 at 37°C. Endothelial cells were prestarved in serum-free medium overnight and then treated with IL-1β, IL-6 and TNF‐α for different hours or different concentrations.

HRGECs stable ATG16L-knockdown and ATG16L-overexpression were constructed using the lentivirus (Jikai, Shanghai, China). HRGECs was first transfected with the lipofectamine 2000, then transfected with 1.5 μL ATG16L shRNA viruses or 2.0 μL ATG16L overexpressing viruses according to the manufacturer’s protocol. The endothelial cells were then switched into ECM containing 2.0 μL polybrene and incubated for an additional 6 h. We detected the infection rate of viruses with a Nikon Eclipse 80i fluorescence microscope (DS-Ri1, Nikon, Shanghai, China). Puromycin (2 µg/mL; Gibco, Thermo Fisher Scientific) was used to select stable virus-infected cells. Thereafter, HRGECs were collected for further experiments.

### Statistical Analysis

GraphPad Prism 5.0 (GraphPad Software, Inc., La Jolla, CA, USA) was used for statistical analysis. Results were expressed as mean ± SD (mean ± SD) from at least three independent experiments. Comparison between and within multiple groups was performed using one-way analysis of variance followed by Student-Newman-Keuls test. *P* values of <0.05 were considered significant.

## Results

### Changes of Histomorphology and Pathology in Kidney Tissues From the Non-CAD and CAD Patients

**Figure 1:**
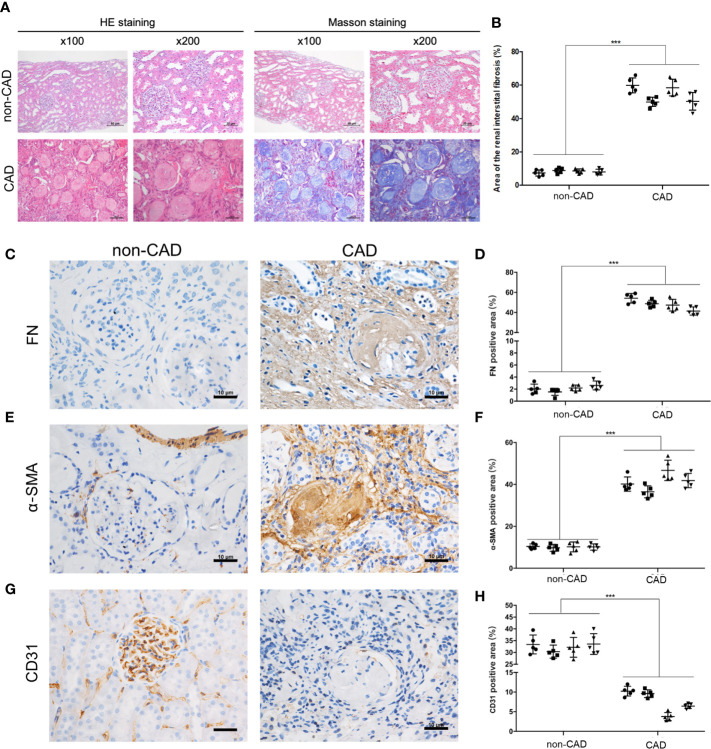
Pathological and morphological changes in kidney tissues from the non-CAD and CAD patients. **(A)** Representative kidney sections from the non-CAD and CAD patients were stained with HE and Masson trichrome (×100, scale bar: 50 μm, ×200, scale bar: 20 μm). **(B)** Semi-quantitative analyses of the degree of fibrosis in 4 non-CAD patients’ and 4 clinical CAD patients’ kidney sections stained with Masson trichrome were performed. Each patient selected 5 different sites of transplanted kidney. (***P< 0.001, CAD vs. the non-CAD group, Student *t* test). **(C, E, G)** Distributions and expressions of FN, α-SMA and CD31 in the CAD and non-CAD group were assessed by IHC staining assays (×400, scale bar: 10 μm). **(D, F, H)** Percentage of the relative abundance of proteins were presented as the mean ± SD values of five independent experiments. Representative images of the kidney tissues of the non-CAD group (n = 4) and CAD group (n = 4) were shown. Each patient selected 5 different sites of transplanted kidney. (***P < 0.001, CAD vs. the non-CAD group, Student *t* test).

In this study, histological examination with hematoxylin-eosin (HE) and Masson’s trichrome staining showed significant renal allograft IF/TA in CAD patients tissues (n = 4) compared to the kidney transplanted patients with stable renal function from our center (non-CAD group, n=4) (**Figures 1A, B**). Sample collection of materials and methods and **Table 1** describes the detailed grouping information for the original features. Immunohistochemistry staining (IHC) of human renal biopsy samples demonstrated a higher expression of α‐SMA, and fibronectin (FN), and remarkably less expression of CD31 in the CAD group (**Figures 1C–H**). CD31 is a marker protein of endothelial cell, and endothelial cell lost its feature CD31 but acquired mesenchymal features such as α‐SMA, FN when EndMT occurs. These results indicated that the expressions of EndMT markers were notably higher in the CAD groups compared to non-CAD group.

### ATG16L Elevated After Kidney Transplantation, but ATG16L Was Downregulated in CAD Patients Comparing to the Non-CAD Patients

**Figure 2:**
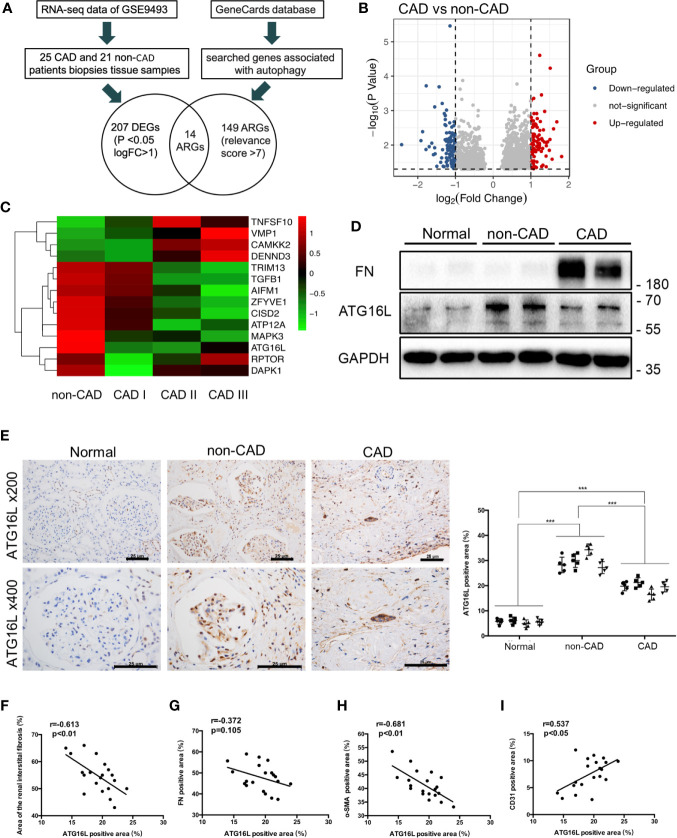
ATG16L expressions in CAD and the non-CAD patients. **(A)** The diagram of the dataset recruitment workflow **(B)** Volcano plot of differential gene expression analysis between non-CAD (n=21) and CAD (n=25) kidney tissues from GEO profiles (GSE9493) **(C)** Heatmap indicating the differential expressions of the 20 selected ARGs. Expression values were presented in red and green to indicate expression upregulation and downregulation, respectively. **(D)** Representative western blot assay results of the protein expressions of ATG16L and FN in normal, non-CAD and CAD patient kidney tissues from our center. **(E)** Representative IHC images of ATG16L expressions in normal, non-CAD and CAD tissue (n=4) (×100, ×200, scale bar: 25 μm). Each patient selected 5 different sites of transplanted kidney. Semi-quantitative analyses of IHC staining were reported (***P < 0.001, vs. the non-CAD group, Student *t* test). **(F)** The correlation analysis between ATG16L and area of renal interstitial fibrosis (r=0.613, *P* < 0.01), **(G)** The correlation analysis between ATG16L and FN (r=0.372, *P* =0.105), **(H)** The correlation analysis between ATG16L and α-SMA (r=0.681, *P* < 0.01), **(I)** The correlation analysis between ATG16L and CD31(r=0.537, *P* < 0.05) were determined using Pearson correlation analysis. (n = 4, biological independent samples. Each patient selected 5 different sites of transplanted kidney).

RNA-seq and clinical data from 25 CAD samples and 21 non-CAD patients’ renal biopsies tissue samples were downloaded from GSE9493. Patients’ information in the database were enrolled at Novartis Institutes for BioMedical Research, Switzerland and processed for histopathology. Histological diagnosis and biopsy classification based on Banff ‘05 criteria. Demographic and clinical data are provided in GEO database (https://www.ncbi.nlm.nih.gov/geo/query/acc.cgi?acc=GSE9493). Schematic diagram of the flow of analyses was as showed in **Figure 2A**. There were 1463 significant differential genes were extracted with *P* value <0.05. Using the criteria for [log2 fold change (log2FC)]> 1, we selected 92 up-regulated and 115 down-regulated differential genes from CAD group compared to the non-CAD group (**Figure 2B**). Then we searched genes associated with autophagy in the GeneCards database. A total of 149 ARGs with relevance score >7 were chosen. There were 14 ARGs included in CAD and non-CAD patients ARGs. Among them, we found that the ATG16L which got highest relevance score gene was downregulated in CAD samples (**Figure 2C**).

In order to avoid the batch effect and population differences, we performed a series of experiments using human specimens from our center to confirm public database results. In the first set, we stained sections of normal, non-CAD and CAD renal samples with an anti-ATG16L antibody, and demonstrated that the expression of ATG16L was significantly reduced in CAD patients compared with non-CAD group (**Figure 2E**). The same conclusion was reached by western blot(WB) assay. The increase of FN represented aggravated fibrosis level of transplant kidney and one of the indicators of CAD. The WB tendency of ATG16L was congruity with the results of our IHC stain verification and consistent with the results in GEO (**Figure 2D**). These suggested that ATG16L expression was instead decreased with progression of CAD. When performing Pearson’s correlation analysis, we found that ATG16L had strong negative correlation in the expression pattern of α‐SMA, FN proteins and positive correlation with CD31 expression. ‘r’ represents the Pearson correlation value and *P* values denote significance of correlation (**Figures 2F–I**).

### Autophagy Was Downregulated in CAD Patients, and Downregulation of MAP1LC3 Predicted Poor Prognosis in CAD Patients

**Figure 3:**
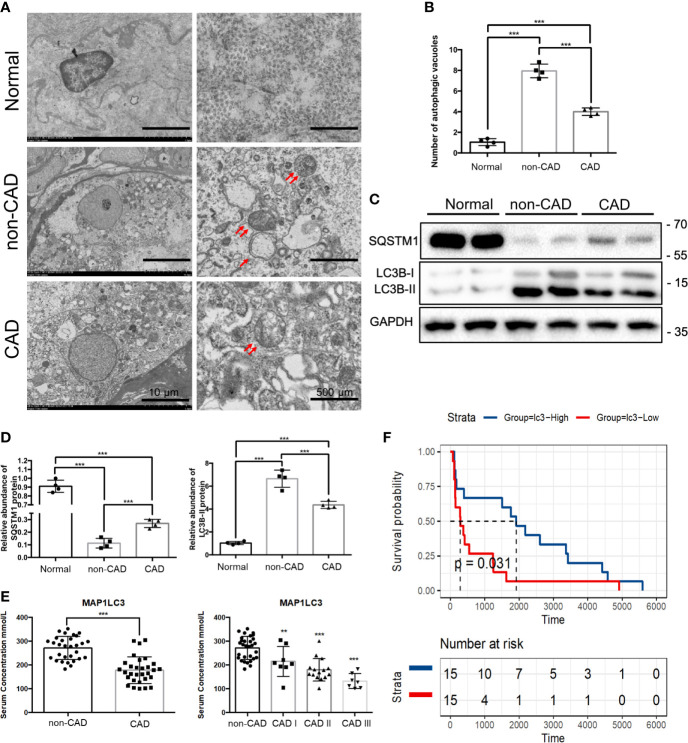
Autophagy, MAP1LC3 expression and prognosis relationship in CAD patients. **(A)** Representative TEM images of autophagic vacuoles (red double arrow) were shown in glomerulus cells from normal, non-CAD and CAD patients (n=4) (scale bar: 10 μm and 500 μm). **(B)** Qualitative analysis of the number of autophagic vacuoles from three groups under TEM were shown. (***P < 0.001, Student *t* test.) **(C)** Representative western blot assay results of the protein expressions of SQSTM1 and LC3BI/II in normal, non-CAD and CAD patient kidney tissues were shown. (n=4) **(D)** Semi-quantitative analysis of Western blot assay results of SQSTM1 and LC3BI/II in normal, non-CAD and CAD patient kidney tissues. (***P < 0.001, Student *t* test.) **(E)** Serum concentrations of MAP1LC3 by *ELISA* assay in 30 non-CAD patients and 30 CAD patients with different degree. (***P < 0.001, **P < 0.01, vs. the non-CAD group, Student *t* test.) **(F)** Survival analysis of different MAPLC3 serum concentrations in 30 CAD patients based on data from *ELISA* assay. (*P*< 0.05, Student *t* test).

In the previous experiment, a number of ARGs that regulate autophagy were identified from the GEO database. Among them, ATG16L is involved in the early step of autophagy, we speculate about the possibility that autophagy could be involved in CAD. To examine the levels of autophagy in human renal allograft tissues, we first found less autophagic vacuoles in CAD group through transmission electron microscope (TEM), compared to non-CAD group (**Figures 3A, B**). Besides the gold standard TEM for monitoring autophagy, decreased levels of SQSTM1 and increase of LC3-II expression reflect the increase of autophagy. Similar trends of protein expression levels of LC3-I (non-lipidation of LC3), LC3-II (lipidation of LC3) and SQSTM1 in CAD group were verified by WB assay as shown in **Figures 3C, D**. P62/SQSTM1 (SQSTM1) is a selective autophagy receptor and is degraded by autophagy. Autophagosomes are associated with the lipidation of the cytosolic form of LC3 and forming of LC3-II. Thus, WB results also suggested that autophagy occurs.

Some studies have shown that there are differences of ARGs in the serum of kidney transplant patients; serum LC3 level probably was the prognostic indicator correlated with severity of the disease (25, 26). To further explore the role of autophagy in CAD procession, we examined the serum LC3 concentration of 30 non-CAD and 30 CAD patients by enzyme linked immunosorbent assay (ELISA). The demographic data presenting the basic features of the patients was in **Table 1**. Distributions of case number, patients sex, patients age, panel reactive antibodies (PRA), immunosuppressive regimen, renal function of the non-CAD and CAD groups were as shown in the **Table 1**. Sex, age, and PRA showed no major differences. There was a significant difference in serum creatinine and blood urea nitrogen (BUN) between the two groups. Then, we tried to determine the prognostic value of autophagy in CAD patients. Through serum ELISA results, we found that the LC3 expression was lower in CAD group. There were also a progressive CAD and a simultaneous decrease in LC3 level (**Figure 3E**). In addition, we divided LC3 into high and low expression groups based on the median, survival analysis, the results revealed LC3 low expression group had shorter allograft dysfunction time (**Figure 3F**). Overall, we speculated that the deficiency of autophagy had a significant association with progression of CAD.

### Downregulation of ATG16L Reduced Autophagy and Promoted Progression of EndMT

**Figure 4:**
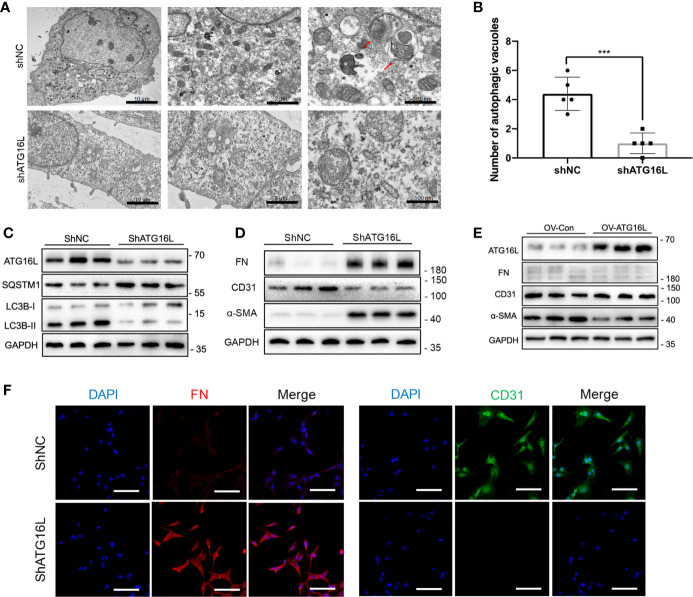
Effects on autophagy and progression of EndMT in HRGECs after knockdown of ATG16L. **(A)** Representative TEM images of autophagic vacuoles (red arrow) were shown in shNC group and shATG16L group (n=5) (scale bar: 10 μm, 2 μm and 500 μm). **(B)** Qualitative analysis of the number of autophagic vacuoles from two groups under TEM were shown. (***P < 0.001, Student *t* test.) **(C)** Western blot assay results of the ATG16L, SQSTM1 and LC3BI/II proteins in HRGECs transfected with shNC or shATG16L. (n=5) **(D)** Expressions of FN, α-SMA and CD31 protein in HRGECs transfected with shNC or shATG16L. (n=5) **(E)** Expressions of ATG16L, FN, α-SMA and CD31 protein in HRGECs overexpressed of ATG16L or not. (n=5) **(F)** Representative images of IF staining of FN and CD31 in HRGECs transfected with shNC or shATG16L. Scale bar: 20 μm (n=5).

To determine whether ATG16L is involved in the change of autophagy, we first transfected HRGECs with ATG16L short hairpin RNA (shRNA). TEM was used to directly demonstrate autophagic vacuoles formation and we found that there are less autophagic vacuoles in cells transfected ATG16L shRNA compared with those transfected with scramble shNC (**Figures 4A, B**). Both of ATG16L protein expression and autophagy activity were also downregulated in knocking down ATG16L group compared with shNC group (**Figure 4C**).

The role of ATG16L-dependent autophagy in progression of EndMT was then investigated. HRGECs were transfected with ATG16L shRNA or shNC. **Figure 4D** showed that ATG16L shRNA transfection could markedly facilitate FN and α-SMA expressions and decrease CD31 expression compared with scramble shNC-transfected HRGECs. In contrast, when the ATG16L were overexpressed in HRGECs, the progression of EndMT did not significantly promote (**Figure 4E**). IHC staining results also confirmed WB trends for CD31 and α-SMA expressions in HRGECs (**Figure 4F**).

### Changes of Renal Interstitial Fibrosis, Urine Protein, Renal Function and Time-Dependent Increases of EndMT in Chronic Rejection Rat Model

**Figure 5:**
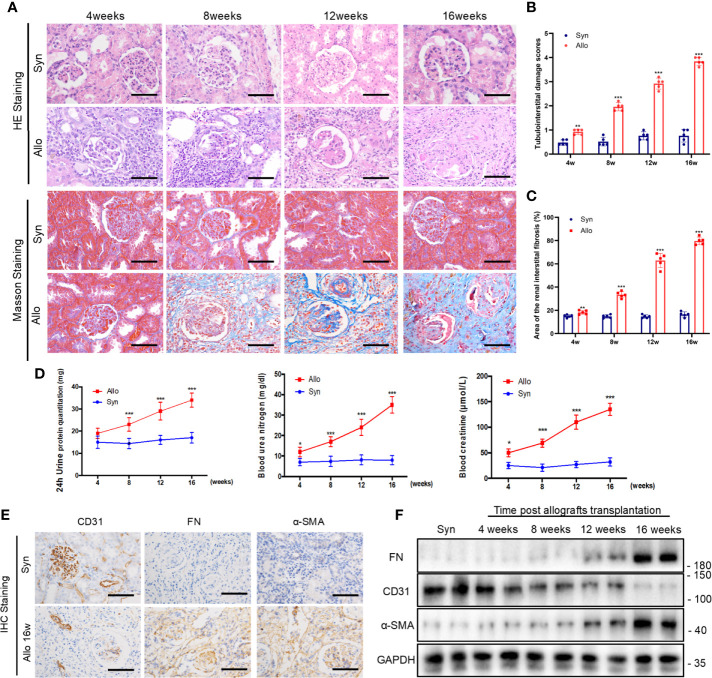
Changes of renal interstitial fibrosis, urine protein, renal function and EndMT in chronic rejection rat model. **(A)** Representative images of HE and Masson’s trichrome staining from kidney tissues of syn and allo groups (scale bar: 25 μm). **(B)** The statistical analyses of tubulointerstitial damage scores in syn and allo group. Data were expressed as the mean ± SD of each group. (n = 5, ***P < 0.001, **P < 0.01, vs. the same weeks syn group, Student *t* test.) **(C)** Semi-quantitative analyses of positive areas of renal interstitial fibrosis were performed from Masson staining assay results. Data were expressed as the mean ± SD of each group (n = 5, ***P < 0.001, **P < 0.01, vs. the same weeks syn group, Student *t* test.). **(D)** Renal function parameters (serum Cr and BUN) and 24h urine protein quantitation in syn and allo group. Values represent the mean ± SD, (n = 5, ***P < 0.001, *P < 0.05, vs. the same weeks syn group, Student *t* test.) **(E)** Representative IHC staining images of EndMT markers (FN, α-SMA and CD31) in kidney sections from syn group and 16 weeks allo group rats (scale bar: 25 μm). **(F)** Representative western blot analysis results of EndMT markers in rat kidney tissues from syn group and different allo group. (n=5).

Next, we investigated the changes of renal interstitial fibrosis, urine protein, renal function and EndMT *in vivo*. Rat renal transplanted models of chronic rejection were established by kidney transplantation from F344 rats to Lewis rats. Renal allografts were taken at weeks 8, 12, and 16 after kidney transplantation. HE staining and Masson staining assays indicated that varying degrees of inflammatory cell infiltration, glomerulosclerosis, tubular atrophy, and interstitial fibrosis after kidney transplantation. The results of Masson staining also showed a large amount of blue-stained collagen fibers in the glomerular and renal interstitium at weeks 8, 12, 16 after kidney transplantation, compared with the syn group (**Figures 5A–C**).

In order to test the renal function of rats with allogeneic transplant, the native right kidneys were extirpated during the kidney transplantation. Four weeks after the resection of right kidney, 24 h urine protein and renal functions including the serum creatinine and BUN deteriorated gradually in allo groups until their death (**Figure 5D**). The outcomes of IHC assay revealed high expressions of FN and α-SMA while CD31 expression reduced in allo groups at 16 weeks (**Figure 5E**). Western blot assay confirmed the outcomes of immunohistochemistry assay and showed that progression of EndMT was intensified with the extension of the time after kidney transplantation (**Figure 5F**).

### Kidney Transplantation Activated Autophagy but Impaired Autophagic Flux in Late Stage of Chronic Rejection Rat Model

**Figure 6:**
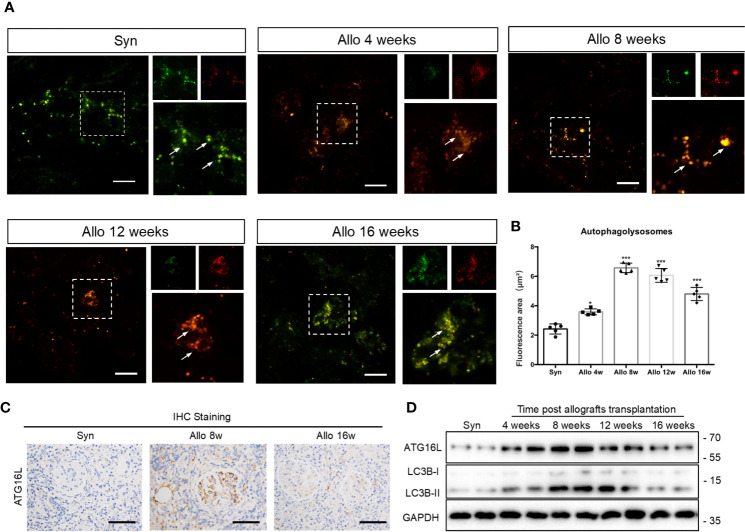
Changes of autophagy activity in chronic rejection rat models. **(A)** Representative images of cortical kidney sections obtained from syn and different periods after kidney transplanted rats injected with AAV-mRFP-GFP-LC3. Arrows indicated the glomerular site. Scale bar: 20 μm **(B)** Semi-quantification analyses of the percentage of autolysosomes (red puncta/total puncta) in the images. The data were presented as mean ± SD (n = 5, ***P < 0.001, *P < 0.05, vs. the syn group). More than 100 cells were quantified for each image in each experiment. **(C)** Representative IHC staining images of ATG16L expressions in syn group, 8 weeks and 16 weeks allo group rats. (n=5) **(D)** Representative western blot assay results of ATG16L and LC3BI/II levels in kidney tissue from syn and allo group rats. (n=5).

LC3, which is considered as a defined marker for autophagy, is critical for autophagosome formation and the activation of autophagic flux. Adeno-associated virus (AAV)-mRFP-GFP-LC3 was injected into the rat renal allograft *in vivo* stereotactically. We create a mRFP-GFP-LC3 model in order to monitor autophagic activity in kidney transplantation. AAV-mRFP-GFP-LC3 is usually used to monitor autophagic flux *in vivo*. GFP signal has higher sensitivity to the acidic conditions of lysosomal lumen than mRFP. Thus, autophagosomes exhibited yellow fluorescence because of the co-location of GFP and mRFP fluorescence indicate and autolysosomes showed red fluorescence. Increase of red fluorescence indicated an accumulation of autolysosomes and activation of autophagy in the kidney. The positive areas of red fluorescence significantly increased at weeks 4, 8,12 and 16 in the allo group compared with the syn group. However, the red puncta weakened rapidly after reaching the peak at 8 and 12 weeks. Changes in the level of autophagy were mainly observed in the glomerular and peripheral small vessel, rather than in the distal tubules or collecting duct (**Figures 6A, B**). These results revealed that autophagy was activated in the early stages of kidney transplantation but not the late stages, and autophagosome clearance was impaired after about 8 weeks. Immunohistochemistry assay demonstrated that the expression of ATG16L was higher in 8 weeks than 16 weeks (**Figure 6C**). Western blot results also showed LC3-II and ATG16L levels peaked at 8 weeks, and then gradually decreased until 16 weeks in rat allograft kidneys after transplantation (**Figure 6D**).

### NF-κB Pathway Was Activated and Promoted EndMT After Knockdown of ATG16L Expression

**Figure 7:**
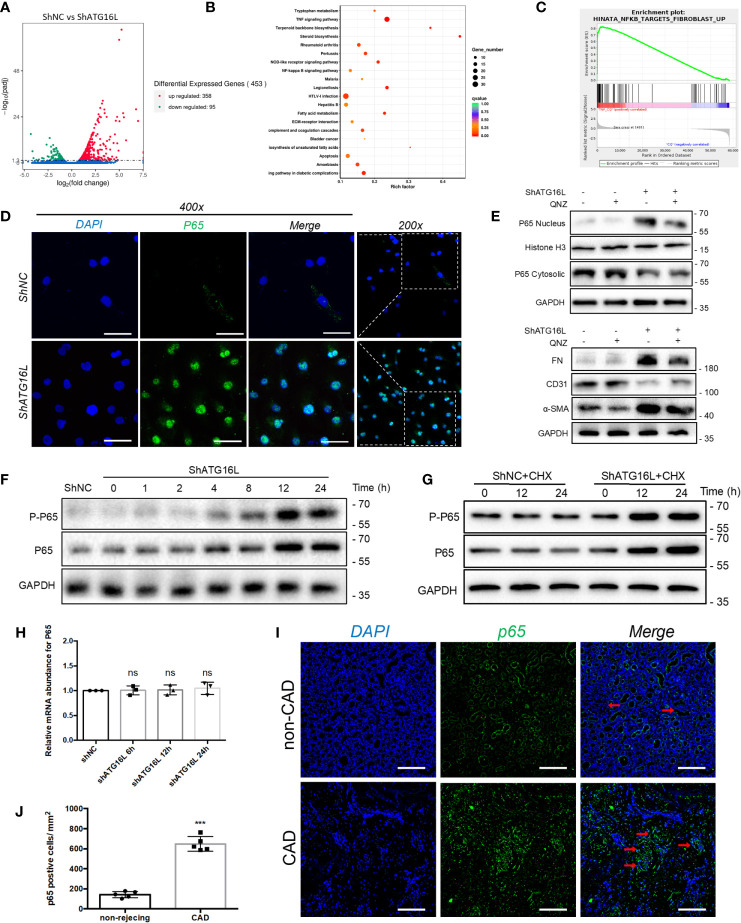
NF-κB pathway activation and effect on EndMT in HRGECs after knockdown of ATG16L **(A)** The RNA sequencing results of differential genes in HRGECs transfected with shNC or shATG16L were plotted by volcano plot. (n=5) **(B)** KEGG pathway analyses of the top 20 KEGG enriched gene pathways. **(C)** GSEA plot of the correlation between knockdown of ATG16L and NF-κB enrichment gene signatures in the RNA sequencing results. **(D)** Representative images of p65 expression and distribution by indirect immunofluorescence staining assay in HRGECs transfected with shNC or shATG16L under confocal microscopy. Scale bar: 50 μm. (n=5) **(E)** Representative western blot results of the effects of NF-κB inhibitor (QNZ) on NF-κB nuclear translocation and EndMT markers (FN, α-SMA and CD31) expressions in HRGECs transfected with shATG16L or not. (n=5) **(F)** Representative western blot results of p65 and p-p65 (Ser536) expressions after knocking down ATG16L for different time. (n=5) **(G)** Representative western blot results of p65 and p-p65 (Ser536) expressions with CHX treatment for different time in shNC and shATG16L group. (n=5) **(H)** The mRNA levels of p65 in HRGECs transfected with shNC or shATG16L. (n=5, NS, not significant, vs. the shNC group, Student *t* test.) **(I)** Representative images of p65 expression and distribution in the non-CAD and CAD allograft renal tissues *via* indirect immunofluorescence staining assay (scale bar: 25 μm). **(J)** Semi-quantitative analysis results for indirect immunofluorescence staining assay of p65 in the non-CAD and CAD allograft renal tissue (n = 4, ***P < 0.001, vs. the non-CAD group, Student *t* test).

In light of the association between ATG16L downregulation and EndMT in chronic rejection rat model and CAD disease, we hypothesized that loss of ATG16L could directly lead to the progression of EndMT. To further explore the potential mechanism by which this progression occurs, we first performed total transcriptome sequencing (RNA-seq) in HRGECs with ShNC or ShATG16L. A total of 453 differential genes (DEGs)were obtained between two groups. Among them, 358 DEGs were up-regulated and 95 were down-regulated (**Figure 7A**). Then 20 pathways were significantly selected by KEGG enrichment. We found that NF-κB pathway was one of the pathways that were significantly activated (**Figure 7B**). Upregulation of the NF-κB pathway genes was also confirmed by Gene set enrichment analysis (GSEA) (**Figure 7C**).

NF-κB is one of the crucial transcription factor for inflammatory signaling pathways, and NF-κB will translocate from cytoplasm to nucleus when it is activated. In order to confirm the NF-κB activation and nuclear translocation, we performed the immunofluorescence staining assay using anti-p65 antibody, and found p65 and the nuclear dye were colocalized which indicated nuclear translocation (**Figure 7D**). In addition, we found that QNZ, a NF-κB signaling pathway inhibitor, significantly inhibited the expression of NF-κB in nucleus, and reversed the expressions of EndMT related proteins induced by knockdown of ATG16L (**Figure 7E**).

WB assay was also carried out to verify the specific mechanism of NF-κB pathway activation. We detected quantification of p65 (total NF-κB) and p-p65 (phosphorylated NF-κB) in HRGECs. WB results showed that p65 level was increased in ShATG16Lgroup compared to ShNC group, while p-p65 followed a similar increasing trend (**Figure 7F**). The increase in p-p65 level in HRGECs after knockdown ATG16L was accompanied by an increase in total p65 protein expression, which likely reflected an additional effect on p65 expression in HRGECs. To detected whether stabilization of NF-κB is due to decreased degradation or increased synthesis, protein synthesis inhibitor cycloheximide (CHX) was added to the cells to inhibit protein synthesis and the NF-κB protein level was analyzed. Western blot results showed that there is continued expression of p65 protein in the presence of CHX (**Figure 7G**). Meanwhile, NF-κB mRNA levels were not yet significantly altered by ShATG16L. The quantitative data for the levels of NF-κB mRNA were as indicated graphically in **Figure 7H**. This suggested that NF-κB nuclear translocation was due to decreased degradation, not increased synthesis after knockdown of ATG16L in HRGECs. To further confirm the expression of NF-κB in human tissue, we performed immunofluorescence staining assay on achieved human renal allograft tissues and found expression level of p65 significant increase in renal allografts of CAD patient (**Figures 7I, J**).

### NF-κB Pathway Activation Induced Cytokines Secretion, Among Them IL-1β, IL-6, and TNF‐α Could Stimulate Progression of EndMT in HRGECs

**Figure 8:**
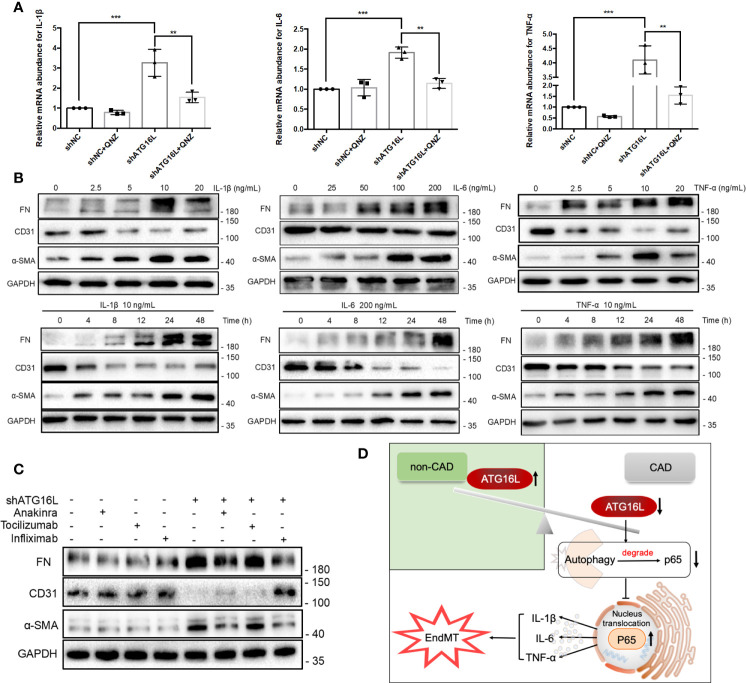
Cytokines induced by NF-κB pathway activation and effects on progression of EndMT in HRGECs. **(A)** The graphs of the results of mRNA abundance of IL-1β, IL-6 or TNF‐α after knocking down ATG16L with or without QNZ. (n = 5, ***P < 0.001, **P < 0.01, vs. the shNC group, Student *t* test.) **(B)** Representative western blotting results of the expressions of FN, CD31, α-SMA and GAPDH in HRGECs after IL-1β, IL-6 or TNF-α treatment for different times or dosage. (n=5) **(C)** Representative western blot results of FN, CD31, α-SMA and GAPDH expressions treated with or without Anakinra, Tocilizumab and Infliximab in HRGECs transfected with shATG16L or not. (n=5) **(D)** A model is proposed to illustrate the mechanisms involved in EndMT induced by loss of ATG16L in the pathogenesis of CAD and transplanted renal interstitial fibrosis.

NF-κB signaling is a master regulator of the inflammatory response. We then explored the soluble factors responsible for shATG16L-induced EndMT by RT-PCR. Among the several cytokines that are relevant to the autophagy defects and inflammatory response, IL-1β, IL-6 and TNF-α, the typical downstream inflammatory factors induced by NF-κB pathway, were significantly elevated when compared with shNC-transfected HRGECs during impairment of autophagy activity (**Figure 8A**). This is in line with our previous findings of inflammatory factors such as TNF-α increase in the rat serum with chronic rejection (27).

To investigate the pathogenesis of EndMT stimulated by IL-1β, IL-6 and TNF-α, we treated the HRGECs with different concentrations of IL-1β (0~20 ng/mL), IL-6 (0~200 ng/mL) and TNF‐α (0~20 ng/mL) for 24 hours. All of IL-1β, IL-6 and TNF-α could upregulate the protein level of FN and α‐SMA with a dose‐dependent decline of the endothelial cell markers CD31 simultaneously. These effects were peaked when HRGECs were treated with 10 ng/mL IL-1β, 200 ng/mL IL-6 or 10 ng/mL TNF‐α, respectively (**Figure 8B**). Time dependencies experiment were also performed to validate the related expressions of EndMT. As shown in **Figure 8B**, IL-1β, IL-6 or TNF-α treatment for 0~48 h could remarkably induced FN and α-SMA expressions and led to the loss of CD31 in HRGECs as the stimulus time increases. To test the effects of IL-1β, IL-6 and TNF-α on EndMT, Anakinra, Tocilizumab and Infliximab treatment were initiated when knocking down ATG16L. Anakinra is an antagonist of interleukin-1 receptor (IL-1R). Tocilizumab is an IL-6R neutralizing antibody that blocks IL-6 from binding to IL-6R. Infliximab is a monoclonal IgG1 antibody that binds specifically to TNF-α and is a TNF-α inhibitor that has been successfully used in the management of inflammatory bowel disease. WB results showed that Anakinra and Infliximab could reduce the EndMT more pronouncedly (**Figure 8C**). These results suggested that the loss of ATG16L was not only reduced autophagy activity but also promoted the secretion of inflammatory cytokines IL-1β, IL-6 and TNF-α by suppressing p65 degradation to activated the NF-κB pathway (**Figure 8D**).

## Discussion

In this present study, we showed that kidney transplantation impaired the autophagic flux, which was associated with an increase in the inflammatory response, EndMT and the severity of renal allografts interstitial fibrosis. Although autophagic activity was temporarily increased at the early stages of allograft transplantation, it gradually decreased due to loss of ATG16L. ATG16L-dependent autophagy, as a cytoprotective process, could attenuate the transplanted kidney interstitial fibrosis *via* the regulation of the EndMT induced by IL-1β, IL-6 and TNF-α. To the best of our knowledge, this is the first study that elucidates the role and dynamic change of autophagic flux in kidney transplantation. In this study, we also investigated the molecular pathophysiologic details underlying the way in which impairment of autophagic flux results in EndMT and renal allograft interstitial fibrosis.

Although many researches have reported that autophagy is involved in the formation and progression of renal interstitial fibrosis, ARGs has not been comprehensively analyzed to explore their clinical value in the progression of chronic renal graft rejection. Hundreds of proteins are associated with the process of autophagy. Given the importance of autophagy in kidney transplantation, it is reasonable to speculate that ARGs hold great promises in prognostic prediction and therapeutic targets. In order to explore the original pathogenetic factors of CAD, we profiled the mRNA expressions of 149 ARGs in the GSE9493 cohort and emphatically analyzed 14 ARGs, which were most associated with autophagy between CAD and non-CAD patients. Among the downregulated ARGs in CAD group, autophagy negative regulatory genes, such as Raptor and MAPK3 (28, 29), were downregulated in CAD group. On the other hand, ATG16L as an up-regulated ARG was scaffold for LC3 lipidation by dynamically localizing to the putative source membranes and promoted autophagic vacuole formation (30). However, some studies have shown that ATG16L was not required for the elongation of the isolation membrane (23). Hence, the association between autophagy-independent or autophagy-dependent role of ATG16L and EndMT was studied.

To investigate the association among ATG16L, autophagy and EndMT in the transplanted kidneys, we knocked down ATG16L by shRNA in HRGECs. Our findings revealed that shATG16L could reduce autophagy level and induce EndMT. Similarly, *in vivo* research and clinical samples also revealed that activation of ATG16L and autophagic flux in the early stage of kidney transplantation, autophagic flux declined in a dynamic range as the fibrotic changes increased. It may be concluded that the reason for EndMT progression is probably due to autophagic flux reduction, and ATG16L-dependent autophagy play a protective role in the progression of EndMT and renal allograft interstitial fibrosis. However, some findings showed that rapamycin-induced autophagy led to activation of EndMT in HCAECs under the anoxic condition (22). These seem to be distinct from our results. We consider that role of autophagy might be different in diverse cell types. It could be cellular context- and upstream regulatory factors- dependent. Giving that ATG16L-dependent autophagy is a means of self-protection when EndMT occur in CAD, it is meaningful to determine the specific mechanism of shATG16L-induced EndMT.

We performed next-generation sequencing from the group of ATG16L knockdown and control group in HRGECs. KEGG pathways and GSEA analysis revealed that NF-κB pathway was one of the most significantly enriched pathways. NF-κB normally binds to IκB, which stabilizes NF-κB in the cytoplasm. When the NF-κB pathway is activated, NF-κB heterodimer p65 translocates to the nucleus and binds to its specific promoter (31). One of the canonical (classical) pathways for NF-κB nuclear translocation is p65 phosphorylation. However, increase in the number of non-canonical pathways have been reported in different diseases. It has also been reported that the suppressed p62/SQSTM1-mediated selective autophagy could reduce NF-κB pathway lysosomal degradation (32), autophagy defect also leads to NF-κB activation because of sustained SQSTM1 expression, which in turn promotes tumorigenesis in mouse models (33). In our study, we observed an increase in p65 and p-p65 protein levels while the mRNA levels were unchanged. In the presence of a protein synthesis inhibitor CHX, accumulation of the p65 protein still continue compared with the absence of inhibitor. We therefore speculated that NF-κB activation induced by knockdown of ATG16L was resulted from reduced protein degradation and this process might be autophagic dependent degradation suppression.

Inflammations are strictly interconnected with important consequences of kidney transplant at clinical and therapeutic level (34). The NF-κB p65 is the most important transcription factor in the regulation of cellular inflammatory factors. In this study, we also found knocking down ATG16L increased cytokine IL-1β, IL-6 and TNF-α through NF-κB pathway. This and other findings agree with the many other studies such as the ones that concluded that loss of ATG16L enhanced endotoxin-induced IL-1β production (35) and TNFAIP3/A20 binds ATG16L1 to control the autophagic response, NF-κB activation (36). Furthermore, the role of inflammatory cytokines on EndMT has been reported in some earlier studies. Nevertheless, it is probable these inflammatory cytokines play a diverse role depending on the underlying pathology and its ambient levels. In diet-induced obesity mouse model endothelial autophagy deficiency induced IL6-dependent EndMT (37). In vascular calcification disease, IL-1β and TNF-α induced EndMT in human primary aortic endothelial cells (38). Our results screened and identified IL-1β, IL-6 and TNF-α as the promoting EndMT inflammatory cytokines in pathological environments of CAD. Herewith, the reciprocal relationships between loss of ATG16L and inflammatory cytokines were researched to underline the possible therapeutic targets to control loss of ATG16L in CAD progression.

Our research has several limitations. The current validation of the effects of ATG16L on EndMT and fibrosis progression were only conducted in knockdown cell line model, the complexity of autophagy alterations *in vivo* and in the development of CAD can hardly be mimicked and underlines the need for endothelial ATG16L conditional knockout mice. We will establish ATG16L knockout mouse renal transplant model for further mechanism study. Our results showed that the loss of ATG16L and autophagy promoted EndMT, QNZ inhibited NF-κB activation and IL-1β, TNF-α monoclonal antibody antagonized specific receptors thereby slowed down the allograft fibrotic process, but it could not completely reverse this outcome. Whether other mechanisms were playing a similar role remains unknown. Additionally, besides endothelial cells, the source of myofibroblasts consists of a variety of cells, including epithelial, fibroblasts and pericytes (39). Whether the loss of ATG16L and autophagy deficiency affect these cells is still inconclusive.

In conclusion, the current studies proved that knockdown of ATG16L was vital for impairment of autophagic flux and EndMT formation induced by IL-1β, IL-6 and TNF‐α in progression of transplanted renal interstitial fibrosis. This function was mediated by NF-κB reducing degradation and pathway activation. In summary, the results of our study provide novel insight into the dynamic relationship of autophagic flux and EndMT. Preventing ATG16L loss and autophagy flux inhibition could be a new option for the treatment and prevention of progression of renal interstitial fibrosis and CAD in kidney transplanted recipients.

## Data Availability Statement

The raw data supporting the conclusions of this article will be made available by the authors, without undue reservation.

## Ethics Statement

The studies involving human participants were reviewed and approved by the local ethics committee of the First Affiliated Hospital of Nanjing Medical University. The patients/participants provided their written informed consent to participate in this study. The animal study was reviewed and approved by the local ethics committee of the First Affiliated Hospital of Nanjing Medical University. Written informed consent was obtained from the owners for the participation of their animals in this study.

## Author Contributions

RT and MG supervised and conceived the project. ZG, CS, and ZW designed and carried out most of the experiments. MZ and SF collected the samples of rats. LS, HC, ZH, JT, XJ, and HY analyzed the data. ZG and ZW made the figures. ZG and RT drafted and revised the paper. All authors contributed to the article and approved the submitted version.

## Funding

This work has been supported by the National Natural Science Foundation of China [grant numbers 82070769, 81870512, 81770751, 81570676, 81470981, 81100532].

## Conflict of Interest

The authors declare that the research was conducted in the absence of any commercial or financial relationships that could be construed as a potential conflict of interest.
